# Metro system in Guangzhou as a hazardous reservoir of methicillin-resistant *Staphylococci*: findings from a point-prevalence molecular epidemiologic study

**DOI:** 10.1038/srep16087

**Published:** 2015-10-29

**Authors:** Yang Peng, Qianting Ou, Dongxin Lin, Ping Xu, Ying Li, Xiaohua Ye, Junli Zhou, Zhenjiang Yao

**Affiliations:** 1 Department of Epidemiology and Health Statistics, Guangdong Pharmaceutical University, Guangzhou 510310, China; 2 Department of Environmental Health and Public Health Laboratory Center, Guangdong Pharmaceutical University, Guangzhou 510310, China

## Abstract

*Staphylococci* are common causes of healthcare-associated and community-associated infections. However, limited data are available on the prevalence, phenotypes and molecular characteristics of *Staphylococci* in metro system around the world. 320 surface samples were collected from the Guangzhou metro system to isolate and characterize *Staphylococci* strains. Of the samples, 75.6% (242/320) were contaminated with *Staphylococci*. The *Staphylococci* isolates, especially the methicillin resistant isolates, were resistance to most of the antibiotics, with 79.8% (193/242) classified as multidrug resistant (MDR) strains. 8 strains of methicillin-resistant *Staphylococcus aureus* (MRSA) carried a range of staphylococcal cassette chromosome *mec* (SCC*mec*) types [I (1), II (3), III (2) and NT (2)]. *Staphylococcus aureus* isolates were classified into several ST types and showed possible cross transmissions of strains from various sources. All MRSA strains were positive for the qac gene, and only one methicillin-susceptible *Staphylococci aureus* (MSSA) strain was positive for the Panton-Valentine leukocidin (PVL) genes. This study demonstrated that environmental surfaces in the Guangzhou metro system may be a hazardous reservoir for transmission of *Staphylococci* to passengers. The resistance to antibiotics and disinfectants observed among isolates was also noteworthy.

*Staphylococcus aureus* is one of the common causes of serious healthcare associated and community associated infections^1^,^2^. Methicillin-resistant *Staphylococcus aureus* (MRSA) strains have spread in many countries and caused various life-threatening infections^3^,^4^,^5^. In addition, an increasing number of reports have shown that coagulase-negative *Staphylococcus* (CoNS), which is considered part of the normal flora in human bodies, has also become a significant conditional pathogen and has displayed a high rate of antibiotic resistance^6^,^7^.

Even more striking is that *Staphylococci* have been isolated and characterized in several non-hospital settings such as buses^8^,^9^, fire stations^10^, universities^11^, and marine beaches^12^. The astonishing survival time of *Staphylococci* on inanimate objects and the high volume of people in public areas facilitate the transmission of *Staphylococci* in such areas. However, there remains a paucity of data regarding the distribution and features of *Staphylococci* circulating in community settings.

The Guangzhou metro system has an average of 5 million passengers daily and the environment is highly enclosed, which creates an ideal setting for the accumulation and transmission of *Staphylococci* among passengers. In this cross-sectional study, the aims were to elucidate the prevalence, antimicrobial susceptibilities, and molecular characteristics of *Staphylococci* strains contaminating metro surfaces in Guangzhou, China.

## Methods

### Environmental sample collection

Surface sampling in the metro system was conducted in November of 2013. Environmental samples were collected from five predetermined locations: hand rails, seats, stanchions, Ticket Vending Machines (TVMs), and escalators. These locations were chosen because they are frequently touched and are more likely to be contaminated with bacteria. The sampling points covered 32 metro stations in 7 lines (line 1, 2, 3, 4, 5, 8 and APM).

Swabs moistened with saline were used to sample surfaces, and the sample area of each swab was approximately 10 cm × 10 cm. To streamline the collection and processing, we used the composite surface sampling method. Each swab was placed into a sterile tube with 7.5% sodium chloride broth and the tubes were transported to the laboratory immediately after sampling.

### Isolation and identification

After 24 hours of incubation at 37 °C, the swabs were transferred to mannitol salt agar plates for another 24 hours of incubation. Colonies were identified as *Staphylococci* through morphology, color and catalase reaction. All *Staphylococci* isolates were further screened for β-haemolysm and were verified as *S. aureus* by tube coagulase testing. The strains were regarded as CoNS if they were negative for coagulase testing. Those *Staphylococci* strains that were positive for the *mecA* gene and/or resistance to cefoxitin were labelled as methicillin-resistant.

### Antimicrobial susceptibility testing

Antibiotic resistance testing was conducted using the Kirby-Bauer disk diffusion method, following the Clinical and Laboratory Standards Institute guidelines^13^. All *Staphylococci* isolates underwent phenotype analysis for antibiotic resistance to 11 antimicrobial agents from 9 antibiotic classes: penicillins (cefoxitin 30 μg, penicillin 10 units); lincosamides (clindamycin 2 μg); ansamycins (rifampicin 5 μg); fluoroquinolones (moxifloxacin 5 μg); aminoglycosides (tobramycins 10 μg, gentamicin 10 μg); sulfonamides (sulfamethoxazole-trimethoprim 25 μg); oxazolidones (linezolid 30 μg); glycopeptides (teicoplanin 30 μg); and macrolides (erythromycin 15 μg). Isolates were classified as multidrug resistant (MDR) if they were non-susceptible to ≥3 antibiotic classes^14^.

### Detection of *mec*A, qac, Panton-Valentine leukocidin, and SCC*mec* typing

All *Staphylococci* isolates were further tested to confirm the presence of the *mec*A gene^12^. *S. aureus* isolates were tested for the presence of the qac gene, an anti-disinfectant gene, and the Panton-Valentine leukocidin (PVL) genes using polymerase chain reaction (PCR) assays^15^.

A multiplex PCR technique was used to confirm and type the staphylococcal cassette chromosome *mec* (SCC*mec*) gene^16^. The results were reported as types I–V, and those isolates that were not type I–V were deemed nontypeable (NT).

### Multilocus sequence typing

The multilocus sequence typing (MLST) PCR assays were carried using previously published primers and conditions^17^. Allelic profiles and sequence types (ST) were assigned using MLST database (http://www. mlst.net). Dendrogram analysis was performed based on ST types to determine the clonal relatedness and potential epidemiologic origin.

### Statistical analysis

Statistical analysis was conducted using Stata 13.0 (College Station, Texas, USA). Data were analyzed using descriptive statistics and χ^2^ tests. A *P* value < 0.05 was considered statistically significant, and all statistical analyses were 2-sided.

## Results

### Identification of Staphylococci isolates

Of the 320 surface samples collected from the stations and carriages in the metro system, a total of 8 samples (2.5%) were MRSA-positive, 28 samples (8.75%) were methicillin-susceptible *Staphylococcus aureus* (MSSA)-positive, 21 samples (6.56%) were methicillin-resistant CoNS (MRCoNS)-positive, 185 samples (57.81%) were methicillin-susceptible CoNS (MSCoNS)-positive, and 78 samples (24.38%) were *Staphylococci*-negative. There were no statistically significant differences in the distributions of isolates between sample locations (χ^2^ = 14.89, *P* = 0.53) (Table 1).

### Antimicrobial susceptibility profiles

Of the 242 *Staphylococci* isolates, 228 (94.21%) displayed resistance to penicillin, 215 (88.84%) to erythromycin, 156 (64.46%) to rifampicin, 109 (45.04%) to trimethoprim, 99 (40.91%) to clindamycin, 76 (31.40%) to gentamicin, 33 (13.64%) to moxifloxicin, 30 (12.40%) to tobramycin, 26 (10.74%) to cefoxitin, 7 (2.89%) to linezolid, and 6 (2.48%) to teicoplanin. The details of the resistance rates among *Staphylococci* tested are summarized in Table 2. The overall MDR rate among *Staphylococci* was 79.75% (193/242). It was 100% for MRSA and MRCoNS isolates, 82.16% (152/185) for MSCoNS isolates, and 42.86% (12/28) for MSSA isolates.

### Molecular characteristics of *S. aureus*

**Figure 1:**
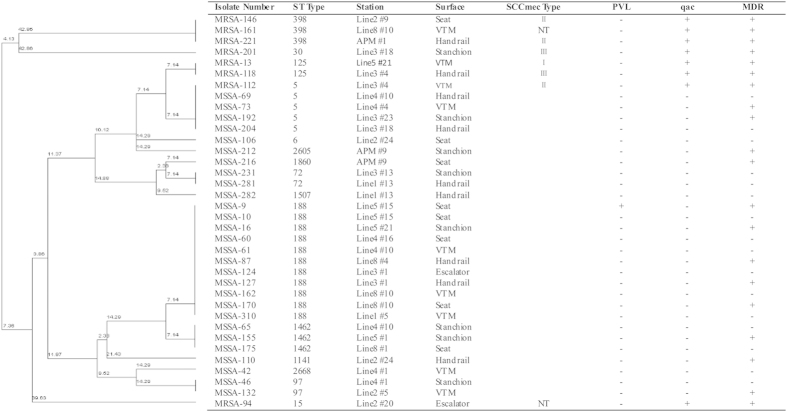
Clonal dendrogram and detailed information on *Staphylococcus aureus* isolates.

Detailed information regarding the molecular features of the *S. aureus* isolates is shown in Fig. 1. Of the eight MRSA isolates, three were classified as SCC*mec* typeII (37.5%), two were type III (25%), one was type I (12.5%), and two were NT (25%). The most predominant ST type among the MRSA strains was ST398 (3/8, 37.5%), followed by ST125 (2/8, 25%), ST5 (1/8, 12.5%), ST15 (1/8, 12.5%) and ST30 (1/8, 12.5%). None of the environmental MRSA isolates carried the PVL genes, and all of them carried the qac gene.

Of the 28 MSSA isolates, ST 188 was the most prevalent ST type (11/28, 39.29%), followed by ST5 (4/28, 14.29%), ST1462 (3/28, 10.71%), ST72 (2/28, 7.14%), ST97 (2/28, 7.14%), ST6 (1/28, 3.57%), ST1141 (1/28, 3.57%), ST1507 (1/28, 3.57%), ST1860 (1/28, 3.57%), ST2605 (1/28, 3.57%) and ST2668 (1/28, 3.57%). Isolate MSSA-9 (ST188) was PVL positive, whereas the remaining MSSA isolates were PVL negative. Unlike the MRSA isolates, none of the MSSA isolate was positive for the qac gene.

## Discussion

To the best of our knowledge, this is the first systematic study to report the occurrence and characteristics of *Staphylococci* strains from environmental surfaces in a metro system of the world. In the current study, 11.25% of the samples were MRSA-positive (2.5%) and/or MSSA-positive (8.75%), whereas the majority (>60%) of the environmental samples were positive for MRCoNS or MSCoNS. The MRSA isolation rate in our work is in similar to that from a Japanese study conducted on trains (2.5% vs 2.3%)^18^. However, considerably higher MRSA detection levels were noted in several previous studies. In two Portuguese studies, 26% (22/85) and 36% (72/199) of sampled buses were tested positive for MRSA contamination^19^,^20^. Additionally, an American study found that 14.8% (35/237) of the surfaces sampled from buses were contaminated with MRSA isolates^8^. The considerable differences in the reported value for MRSA prevalence could be affected by factors such as limited sampling locations, varying sampling techniques, and different regional hygiene measures.

The findings of drug resistance in this study are also noteworthy. The levels of resistance to some common antibiotics, such as penicillin, erythromycin and rifampicin, are alarming, especially among methicillin resistant strains. In addition, some isolates were even resistant to teicoplanin and linezolid, both of which are final effective agents against *Staphylococci* infections^21^. Even more intriguing is that most of the *Staphylococci* isolates displayed the characteristics of multidrug resistance, suggesting that they are more likely to have originated from healthcare-associated settings.

The results of the analyses of molecular features further broadened our insights into *Staphylococci* in a non-hospital environment. ST 188, the most predominant ST type of MSSA in our study, is widely disseminated in China and originated from community and hospital settings^22^,^23^. Some of the isolates, such as MSSA-16, MSSA-87, MSSA-127 and MSSA-170, have same molecular characteristics, suggesting they may represent a single clone. It is possible that the strains were transferred to the contaminated surfaces by discharged patients and/or healthcare workers who carried the recent epidemic strains in Guangzhou, although the sources of these strains are unclear. In contrast, ST398 and ST97 were also observed in the present study, and they have been reported as typical animal clones in many countries^24^,^25^,^26^,^27^. The appearance of these clones in an urban environment is rarely reported and, thus, we cannot rule out the possibility that those five ST398 and ST97 isolates were transmitted by passengers who have close contacts with animals or meats. Most of the MRSA isolates were typed as SCC*mec* I–III, and all of them were negative for the PVL genes; both of these features are indicators of hospital-acquired isolates. This phenomenon is in agreement with the findings of the drug resistance testing. A high frequency of the anti-disinfectant qac gene was discovered among all MRSA isolates in our study, and this gene was previously detected in airborne *Staphylococci* isolates from Shanghai metro stations^15^. Hence, the efficiency of sterilization processes in metro systems should be improved by reselection of suitable disinfectants.

In conclusion, our study demonstrated that the frequently touched surfaces in metro system may be a reservoir for *Staphylococci* transmissions and the resistance to antimicrobials and disinfectants is prevalent, especially among methicillin resistant strains. Cross transmissions of *Staphylococci* isolates from various sources, including hospitals, communities and livestock, are possible. More stringent infection control and surveillance measures are urgently needed.

## Additional Information

**How to cite this article**: Peng, Y. *et al.* Metro system in Guangzhou as a hazardous reservoir of methicillin-resistant *Staphylococci*: findings from a point-prevalence molecular epidemiologic study. *Sci. Rep.* **5**, 16087; doi: 10.1038/srep16087 (2015).

## Figures and Tables

**Table 1:** Distribution of isolates among different sample locations.

Location	MRSA n (%)	MSSA n (%)	MRCoNS n (%)	MSCoNS n (%)	*Staphylococci*(–) n (%)	Total
Seats	1 (1.56)	7 (10.94)	3 (4.69)	33 (51.56)	20 (31.25)	64
Escalators	1 (1.56)	1 (1.56)	7 (10.94)	36 (56.25)	19 (29.69)	64
Hand rails	2 (3.13)	7 (10.94)	3 (4.69)	36 (56.25)	16 (25.00)	64
Stanchions	1 (1.56)	7 (10.94)	4 (6.25)	39 (60.94)	13 (20.31)	64
VTMs	3 (4.69)	6 (9.38)	4 (6.25)	41 (64.06)	10 (15.63)	64
Total	8 (2.50)	28 (8.75)	21 (6.56)	185 (57.81)	78 (24.38)	320

TVMs, Ticket Vending Machines.

**Table 2:** Antibiotic resistance rates for tested *Staphylococci* [n (%)].

Drug agent	MRSA n (%)	MSSA n (%)	MRCoNS n (%)	MSCoNS n (%)	Total n (%)
CEF	6 (75.00)	0 (0.00)	20 (95.24)	0 (0.00)	26 (10.74)
CLI	7 (87.50)	8 (28.57)	21 (100.00)	107 (57.84)	99 (40.91)
RIF	5 (62.50)	6 (21.43)	15 (71.43)	60 (32.43)	156 (64.46)
MOX	4 (50.00)	4 (14.29)	11 (52.38)	14 (7.57)	33 (13.64)
TOB	5 (62.50)	8 (28.57)	8 (38.10)	9 (4.86)	30 (12.40)
TRI	5 (62.50)	5 (17.86)	18 (85.71)	81 (43.78)	109 (45.04)
PEN	8 (100.00)	24 (85.71)	21 (100.00)	175 (94.59)	228 (94.21)
LIN	2 (25.00)	0 (0.00)	5 (23.81)	0 (0.00)	7 (2.89)
TEI	1 (12.50)	0 (0.00)	4 (19.05)	1 (0.54)	6 (2.48)
ERY	7 (87.50)	14 (50.00)	21 (100.00)	173 (93.51)	215 (88.84)
GEN	6 (75.00)	6 (21.43)	12 (57.14)	52 (28.11)	76 (31.40)

CEF, cefoxitin; CLI, clindamycin; RIF, rifampicin; MOX, moxifloxicin; TOB, tobramycin; TRI, trimethoprim; PEN, penicillin; LIN, linezolid;

TEI, teicoplanin; ERY, erythromycin; GEN, gentamicin.

## References

[b1] Sasan M., Donyadide N., Askari E. & Naderi-Nasab M. Invasive community-acquired Staphylococcus aureus among pediatric population of Eastern Iran. Iranin J Microbiol 6, 84–86 (2014). PMC4281665 25705357

[b2] Yang Z. *et al.* Proportions of Staphylococcus aureus and methicillin-resistant Staphylococcus aureus in patients with surgical site infections in mainland China: a systematic review and meta-analysis. PLoS One 10, e0116079 (2015). 25602284 10.1371/journal.pone.0116079 PMC4300093

[b3] Immergluck L. C. *et al.* Methicillin-resistant Staphylococcus aureus colonization among pediatric health care workers from different outpatient settings. Am J Infect Control 41, 841–843 (2013). 23433983 10.1016/j.ajic.2012.11.014 PMC3759650

[b4] Zhang H. *et al.* A multicentre study of meticillin-resistant Staphylococcus aureus in acute bacterial skin and skin-structure infections in China: Susceptibility to ceftaroline and molecular epidemiology. Int J Antimicrob Agents 45, 347–350 (2015). 25649348 10.1016/j.ijantimicag.2014.12.014

[b5] Yaw L. K., Robinson J. O. & Ho K. M. A comparison of long-term outcomes after meticillin-resistant and meticillin-sensitive Staphylococcus aureus bacteraemia: an observational cohort study. Lancet Infect Dis 14, 967–975 (2014). 25185461 10.1016/S1473-3099(14)70876-X

[b6] Lerbech A. M. *et al.* Antibiotic exposure in a low-income country: screening urine samples for presence of antibiotics and antibiotic resistance in coagulase negative staphylococcal contaminants. PLoS One 9, e113055 (2014). 25462162 10.1371/journal.pone.0113055 PMC4251977

[b7] Ma X. X., Wang E. H., Liu Y. & Luo E. J. Antibiotic susceptibility of coagulase-negative staphylococci (CoNS): emergence of teicoplanin-non-susceptible CoNS strains with inducible resistance to vancomycin. J Med Microbiol 60, 1661–1668 (2011). 21799199 10.1099/jmm.0.034066-0

[b8] Lutz J. K. *et al.* Methicillin-resistant Staphylococcus aureus in public transportation vehicles (buses): another piece to the epidemiologic puzzle. Am J Infect Control 42, 1285–1290 (2014). 25465258 10.1016/j.ajic.2014.08.016

[b9] Stepanovic S., Cirkovic I., Djukic S., Vukovic D. & Svabic-Vlahovic, M. Public transport as a reservoir of methicillin-resistant staphylococci. Lett Appl Microbiol 47, 339–341 (2008). 19241530 10.1111/j.1472-765x.2008.02436.x

[b10] Roberts M. C. & No D. B. Environment surface sampling in 33 Washington State fire stations for methicillin-resistant and methicillin-susceptible Staphylococcus aureus. Am J Infect Control 42, 591–596 (2014). 24837108 10.1016/j.ajic.2014.02.019

[b11] Roberts M. C., Soge O. O., No D., Helgeson S. E. & Meschke J. S. Characterization of Methicillin-resistant Staphylococcus aureus isolated from public surfaces on a university campus, student homes and local community. J Appl Microbiol 110, 1531–1537 (2011). 21447018 10.1111/j.1365-2672.2011.05017.x

[b12] Soge O. O., Meschke J. S., No D. B. & Roberts M. C. Characterization of methicillin-resistant Staphylococcus aureus and methicillin-resistant coagulase-negative Staphylococcus spp. isolated from US West Coast public marine beaches. J Antimicrob Chemother 64, 1148–1155 (2009). 19837712 10.1093/jac/dkp368 PMC2782242

[b13] Clinical and Laboratory Standards Institute. Performance standards for antimicrobial susceptibility testing: 23rd informational supplement. CLSI 33, M100–S23 (2013).

[b14] Magiorakos A. P. *et al.* Multidrug-resistant, extensively drug-resistant and pandrug-resistant bacteria: an international expert proposal for interim standard definitions for acquired resistance. Clin Microbiol Infect 18, 268–281 (2012). 21793988 10.1111/j.1469-0691.2011.03570.x

[b15] Zhou F. & Wang Y. Characteristics of antibiotic resistance of airborne Staphylococcus isolated from metro stations. Int J Environ Res Public Health 10, 2412–2426 (2013). 23765189 10.3390/ijerph10062412 PMC3717744

[b16] Zhang K., McClure J. A., Elsayed S., Louie T. & Conly J. M. Novel multiplex PCR assay for characterization and concomitant subtyping of staphylococcal cassette chromosome mec types I to V in methicillin-resistant Staphylococcus aureus. J Clin Microbiol 43, 5026–5033 (2005). 16207957 10.1128/JCM.43.10.5026-5033.2005 PMC1248471

[b17] Enright M. C., Day N. P., Davies C. E., Peacock S. J. & Spratt B. G. Multilocus sequence typing for characterization of methicillin-resistant and methicillin-susceptible clones of Staphylococcus aureus. J Clin Microbiol 38, 1008–1015 (2000). 10698988 10.1128/jcm.38.3.1008-1015.2000 PMC86325

[b18] Iwao Y. *et al.* Isolation and molecular characterization of methicillin-resistant Staphylococcus aureus from public transport. Microbiol Immunol 56, 76–82 (2012). 22040019 10.1111/j.1348-0421.2011.00397.x

[b19] Simoes R. R. *et al.* High prevalence of EMRSA-15 in Portuguese public buses: a worrisome finding. PLoS One 6, e17630 (2011). 21407807 10.1371/journal.pone.0017630 PMC3047573

[b20] Conceicao T., Diamantino F., Coelho C., de Lencastre H. & Aires-de-Sousa M. Contamination of public buses with MRSA in Lisbon, Portugal: a possible transmission route of major MRSA clones within the community. PLoS One 8, e77812 (2013). 24223124 10.1371/journal.pone.0077812 PMC3819345

[b21] Kuti J. L., Kiffer C. R., Mendes C. M. & Nicolau D. P. Pharmacodynamic comparison of linezolid, teicoplanin and vancomycin against clinical isolates of Staphylococcus aureus and coagulase-negative staphylococci collected from hospitals in Brazil. Clin Microbiol Infect 14, 116–123 (2008). 18076672 10.1111/j.1469-0691.2007.01885.x

[b22] Qiao Y. *et al.* Clinical and molecular characteristics of invasive community-acquired Staphylococcus aureus infections in Chinese children. BMC Infect Dis 14, 582 (2014). 25377600 10.1186/s12879-014-0582-4 PMC4225039

[b23] He W. *et al.* Population structure and characterisation of Staphylococcus aureus from bacteraemia at multiple hospitals in China: association between antimicrobial resistance, toxin genes and genotypes. Int J Antimicrob Agents 42, 211–219 (2013). 23871455 10.1016/j.ijantimicag.2013.04.031

[b24] Silva N. C. *et al.* Methicillin-resistant Staphylococcus aureus of lineage ST398 as cause of mastitis in cows. Lett Appl Microbiol 59, 665–669 (2014). 25236329 10.1111/lam.12329

[b25] Yan X. *et al.* Staphylococcus aureus ST398 from slaughter pigs in northeast China. Int J Med Microbiol 304, 379–383 (2014). 24418357 10.1016/j.ijmm.2013.12.003

[b26] Gomez-Sanz E. *et al.* Detection, molecular characterization, and clonal diversity of methicillin-resistant Staphylococcus aureus CC398 and CC97 in Spanish slaughter pigs of different age groups. Foodborne Pathog Dis 7, 1269–1277 (2010). 20677918 10.1089/fpd.2010.0610

[b27] Hata E. *et al.* Genetic variation among Staphylococcus aureus strains from bovine milk and their relevance to methicillin-resistant isolates from humans. J Clin Microbiol 48, 2130–2139 (2010). 20392913 10.1128/JCM.01940-09 PMC2884479

